# Supplementary figures and images for: Harmonised culture procedures minimise but do not eliminate mesenchymal stromal cell donor and tissue variability in a decentralised multicentre manufacturing approach
Source: Stem Cell Res Ther. 2023 May 4;14:120. doi: 10.1186/s13287-023-03352-1 (PMC10161493; doi:10.1186/s13287-023-03352-1)

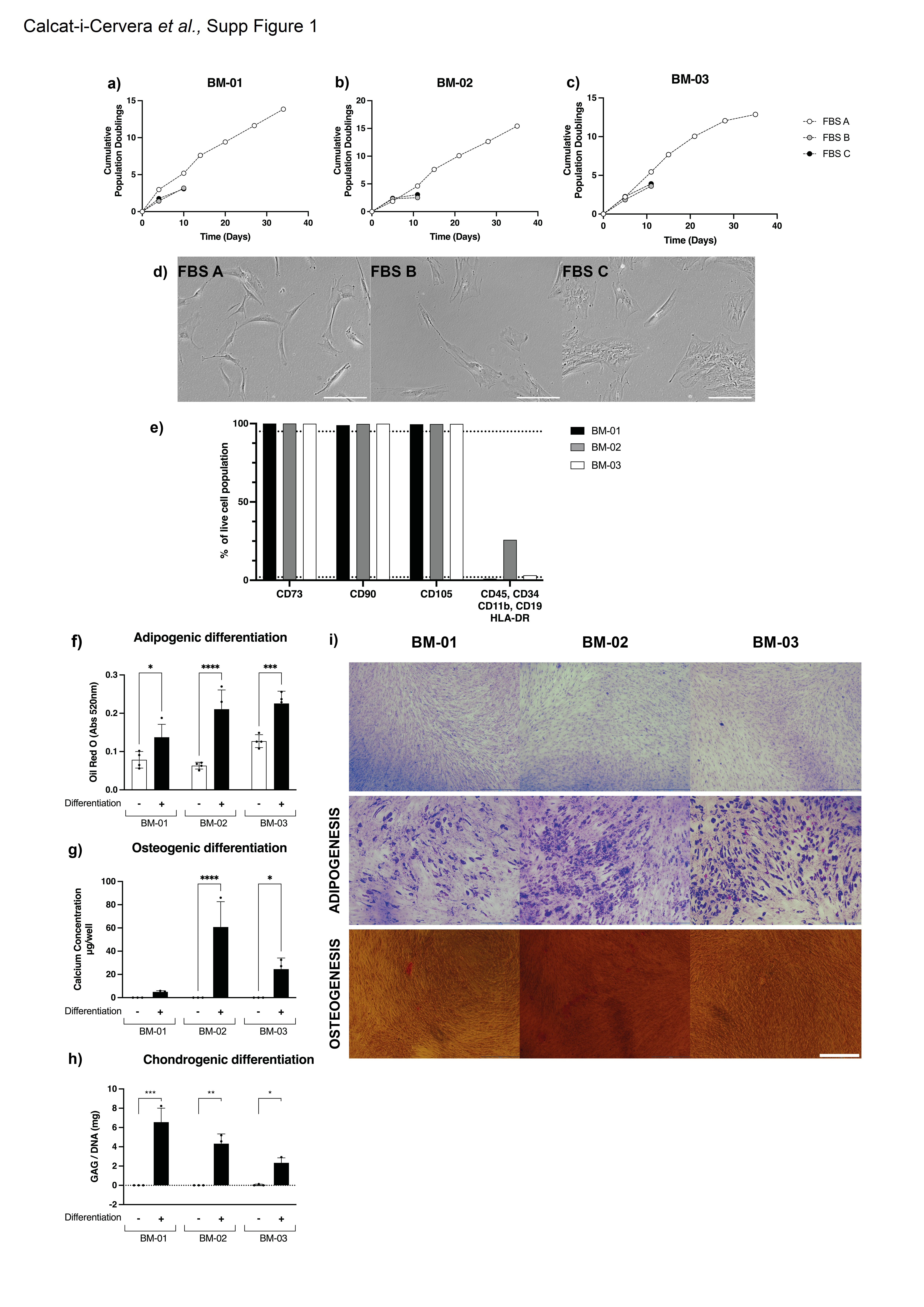

Supplement: Supplementary file 1 — Additional file 1: Supplementary Fig. S1. Cell Culture Harmonisation: Serum Screen.Population doubling times andphase contrast images of three BM-MSCs donors showed that exclusively FBS A supported cell growth and fibroblast-like morphology. Therefore, further experiments were carried out using serum A.Flow cytometry confirmed the expression of positive surface antigensand lack of negative markersin two out of three populations grown with FBS A.BM-MSC cultures were induced to differentiate into adipocyteswhile undifferentiated cultures served as control. Images of Oil Red O are shown in paneland quantification of Oil Red O stain retention in panel. Both show an increase in lipid content in the majority of adipogenic differentiated cultures.Osteogenic differentiated cultures showed presence of calcium in the extracellular matrix with Alizarin Red staining.Quantification of extracted calcium from osteogenically differentiated BM-MSC showed more than 1 µg of calcium per well in all differentiated cultures.Quantification of sulphated glycosaminoglycansshowed significantly increased levels in differentiated cultures, confirming their mesodermal differentiation abilities. Data displayed as mean ± SD, N=3. Two-Way ANOVA with Bonferroni’s multiple comparison corrections, * = p < 0.05, ** = p < 0.001, ** = p < 0.0001, **** = p < 0.00001. Pictures taken at 40X; scale bar 500 µm. [file 13287_2023_3352_MOESM1_ESM.jpg]

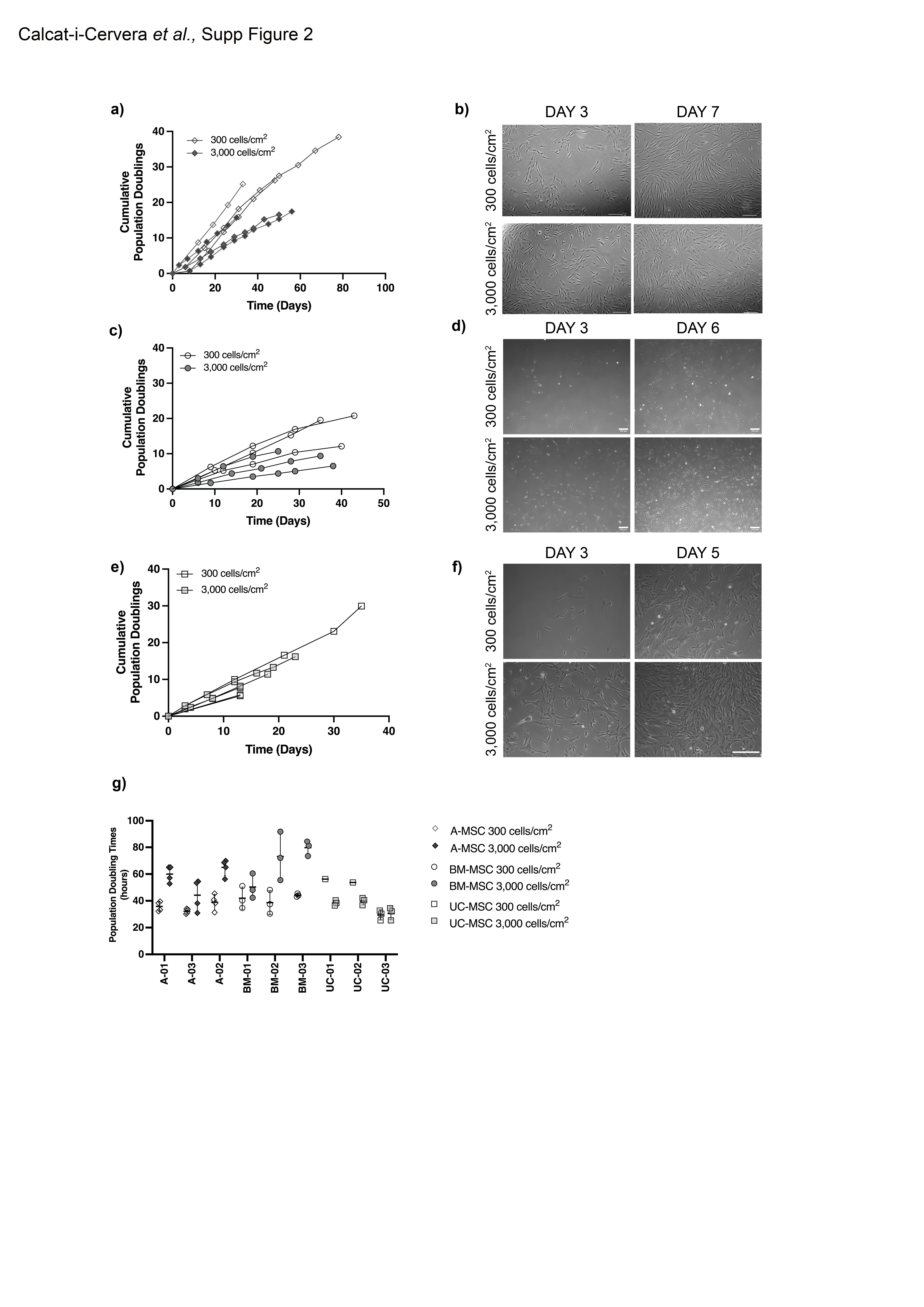

Supplement: Supplementary file 2 — Additional file 2: Supplementary Fig. S2. Cell Culture Harmonisation: Seeding Density. Comparison between seeding density confirmed differences in cell source. Cumulative population doublings were calculated by culturing MSCs at 300and 3,000 cells/cm2in all three different sites.A-MSC andBM-MSC showed a rapid increase in cumulative doublings when seeded at a lower density versus at high density after the same period in culture.UC-MSC conversely had increased cumulative doublings when seeded at higher density.When comparing population doubling times, A-MSC and BM-MSC had prolonged kinetics when grown at 3,000 cells/cm2 whereas UC-MSC divided faster at 3,000 cells/cm2.Representative phase contrast images of MSCs. Data displayed as mean ± SD, N=3. Pictures taken at 40X. [file 13287_2023_3352_MOESM2_ESM.jpg]

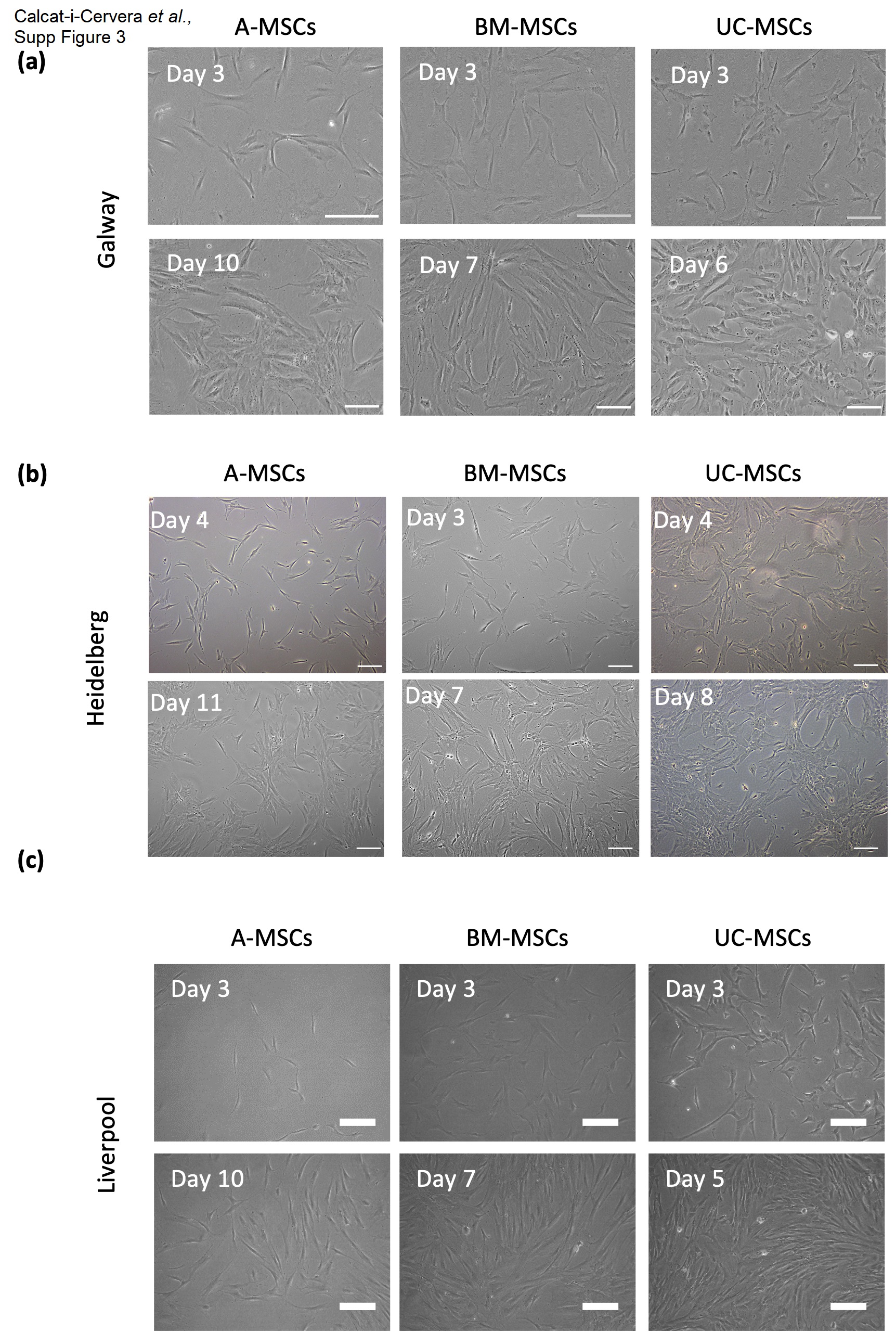

Supplement: Supplementary file 3 — Additional file 3: Supplementary Fig. S3. Representative phase contrast images of MSCs in all sites at earlyand latestages of culture. Pictures taken at 100X; scale bar 200 µm. [file 13287_2023_3352_MOESM3_ESM.jpg]

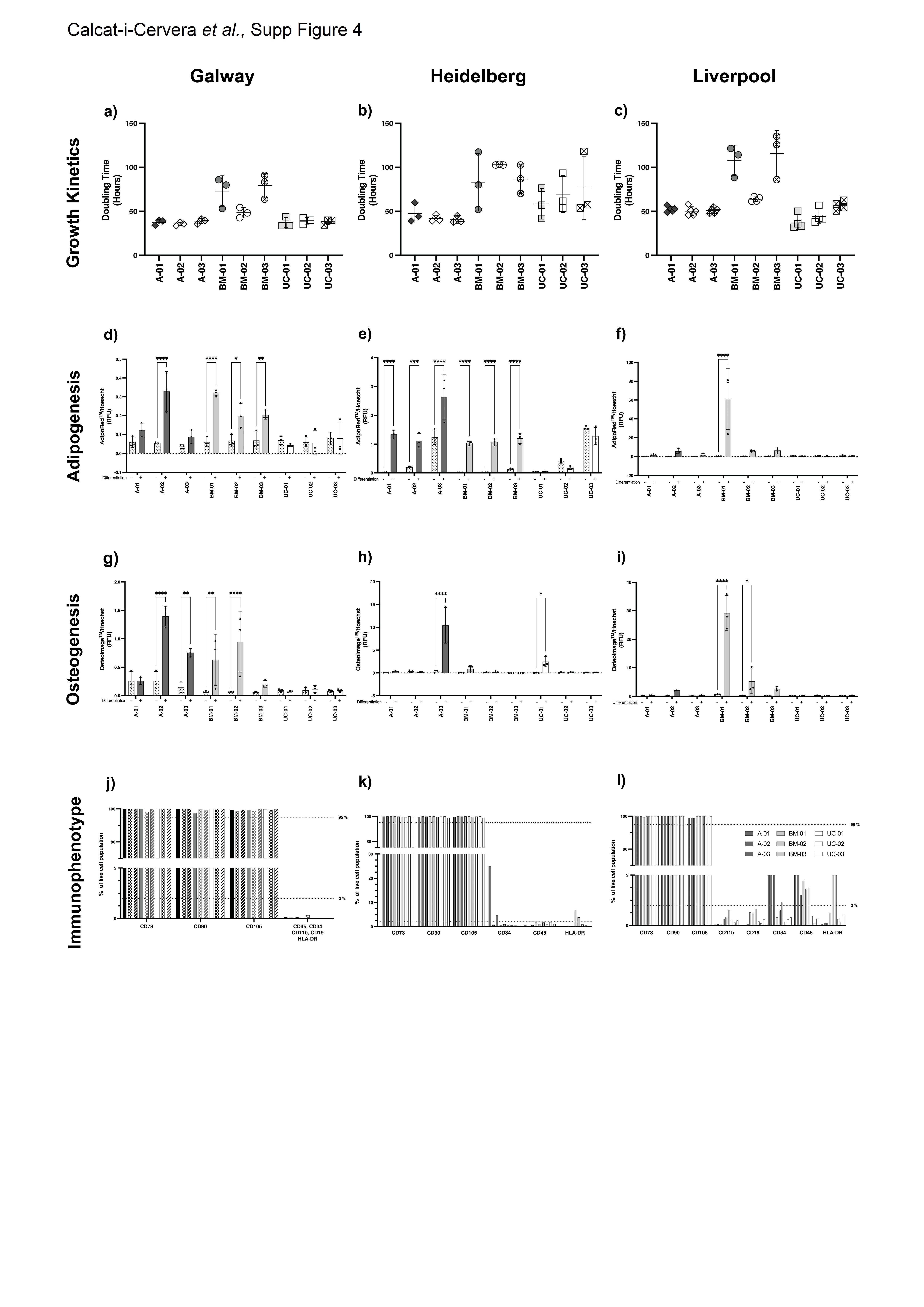

Supplement: Supplementary file 4 — Additional file 4: Supplementary Fig. S4. Biological comparison: donor-by-donor breakdown of doubling times, immunophenotype, differentiation results and phase contrast images of the differentiation. Figuresshow the individual doubling times per each donor in all sites. The three dots within a single donor represent the doubling times from three consecutive passages. Across laboratories, A- and UC- showed stable proliferation rates when looking at individual donors. Greater differences were seen in BM- in terms of donor-to-donor variability, although each donor behaved similarly regardless of manufacturing site. In terms of committing to mesodermal lineages, high variability of induction was seen across laboratories. Broadly, A- and BM- donors were able to undergo adipogenesis in two sites, apart from one particular donor that showed induction in all laboratories. Negligible levels of adipogenic differentiation were seen in UC-MSC cultures. Similarly, A- and BM-MSCs were able to undergo osteogenic differentiation in two out of three sites, albeit not all donors and at remarkable different rates; exclusively one UC-MSCs in one site showed moderate levels of osteogenesis. Assessment of surface antigen expression confirmed >95% levels of CD73, CD90 and CD105 in all donors across sites. However, two preparations of A-MSC showed higher than 2% levels of CD34 in two and CD45 in one site. Importantly, these were the same donors. Data displayed as mean ± SD, N=3, n=3. One-Way ANOVA with Tukey’s multiple comparison corrections, * = p < 0.05, ** = p < 0.001, ** = p < 0.0001, **** = p < 0.00001. [file 13287_2023_3352_MOESM4_ESM.jpg]

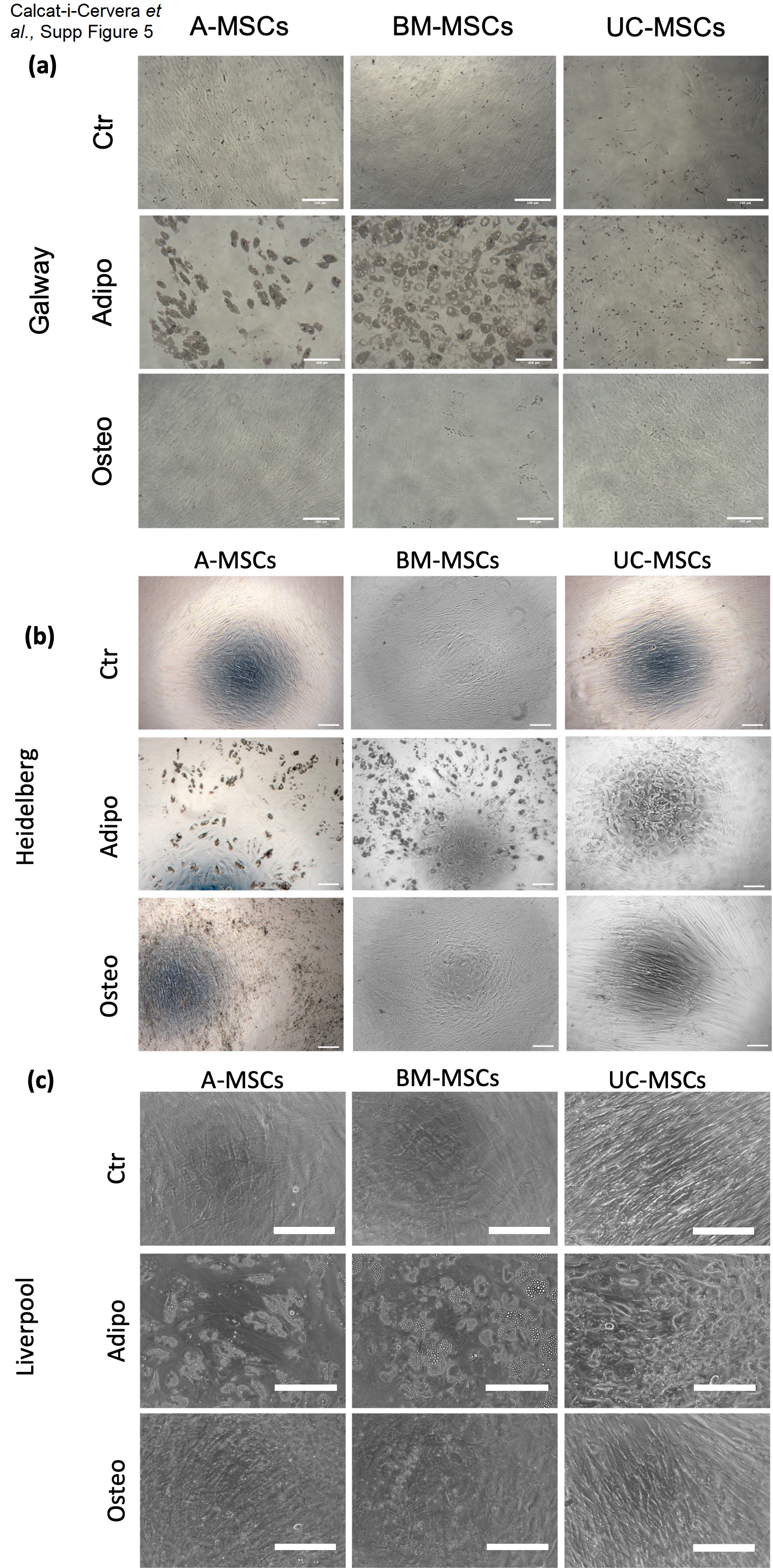

Supplement: Supplementary file 5 — Additional file 5: Supplementary Fig. S5. Representative phase contrast images of MSC at the end of the adipogenicand osteogenicdifferentiation procedure in comparison with undifferentiated culturesin each site. Pictures taken at 100X; scale bar 200 µm. [file 13287_2023_3352_MOESM5_ESM.jpg]

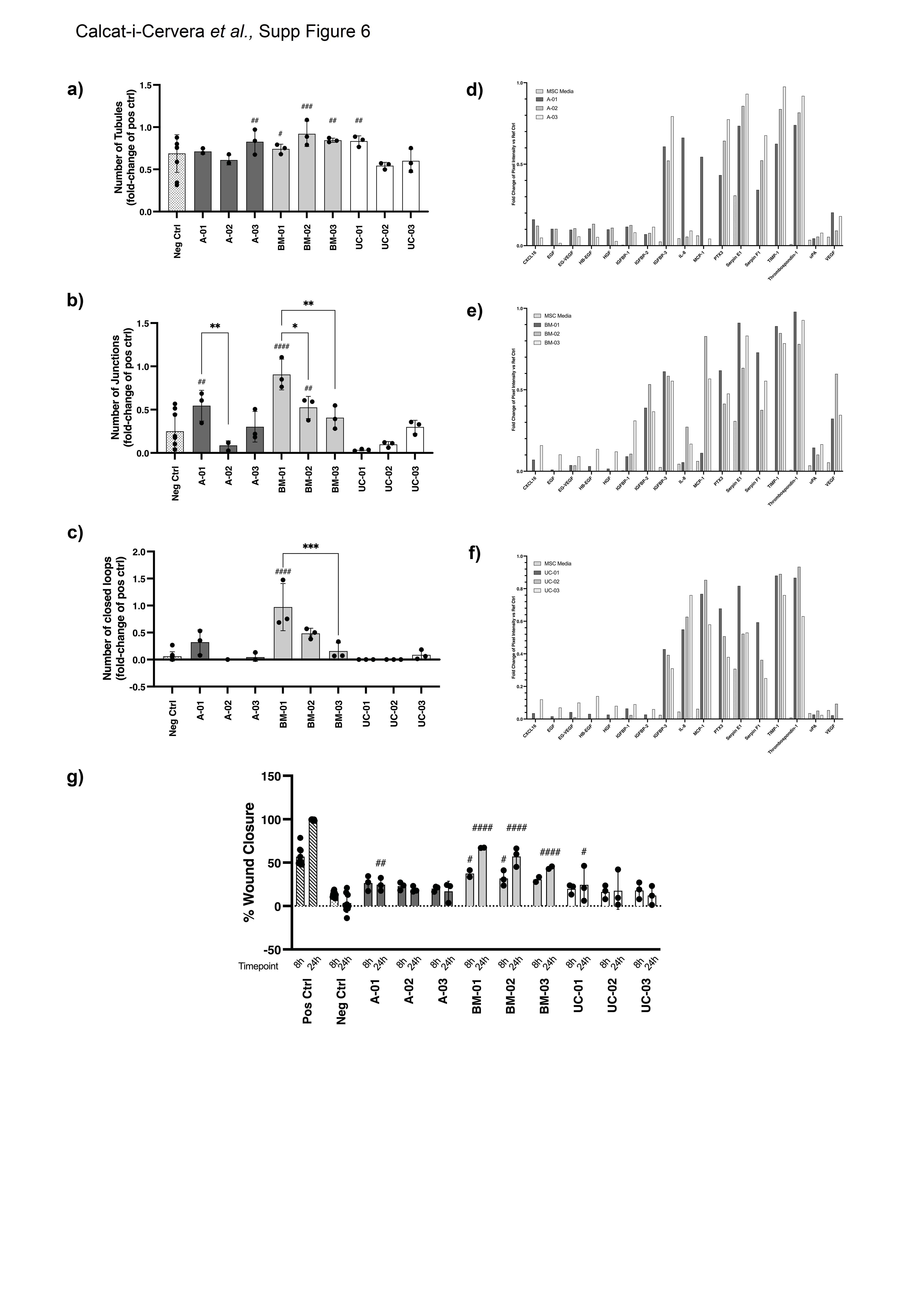

Supplement: Supplementary file 6 — Additional file 6: Supplementary Fig. S6. Angiogenic and wound healing properties of MSCs listed by donor.Number of tubules, junctionsand closed loopsgenerated by each donor. Data expressed as a fold-change of the positive control; mean ± SD, n = 3.Differential angiogenic proteomic profile detected for A-, BM-and UC-MSCs. Data expressed as fold change of the internal reference spots.Wound closure induced by each donor per cell source at 8 and 24 hours. Data displayed as mean ± SD, n = 3. Two-Way ANOVA with Tukey’s multiple comparison corrections, * = p < 0.05, ** = p < 0.001, ** = p < 0.0001, **** = p < 0.00001. # Significance relative to negative control. [file 13287_2023_3352_MOESM6_ESM.jpg]

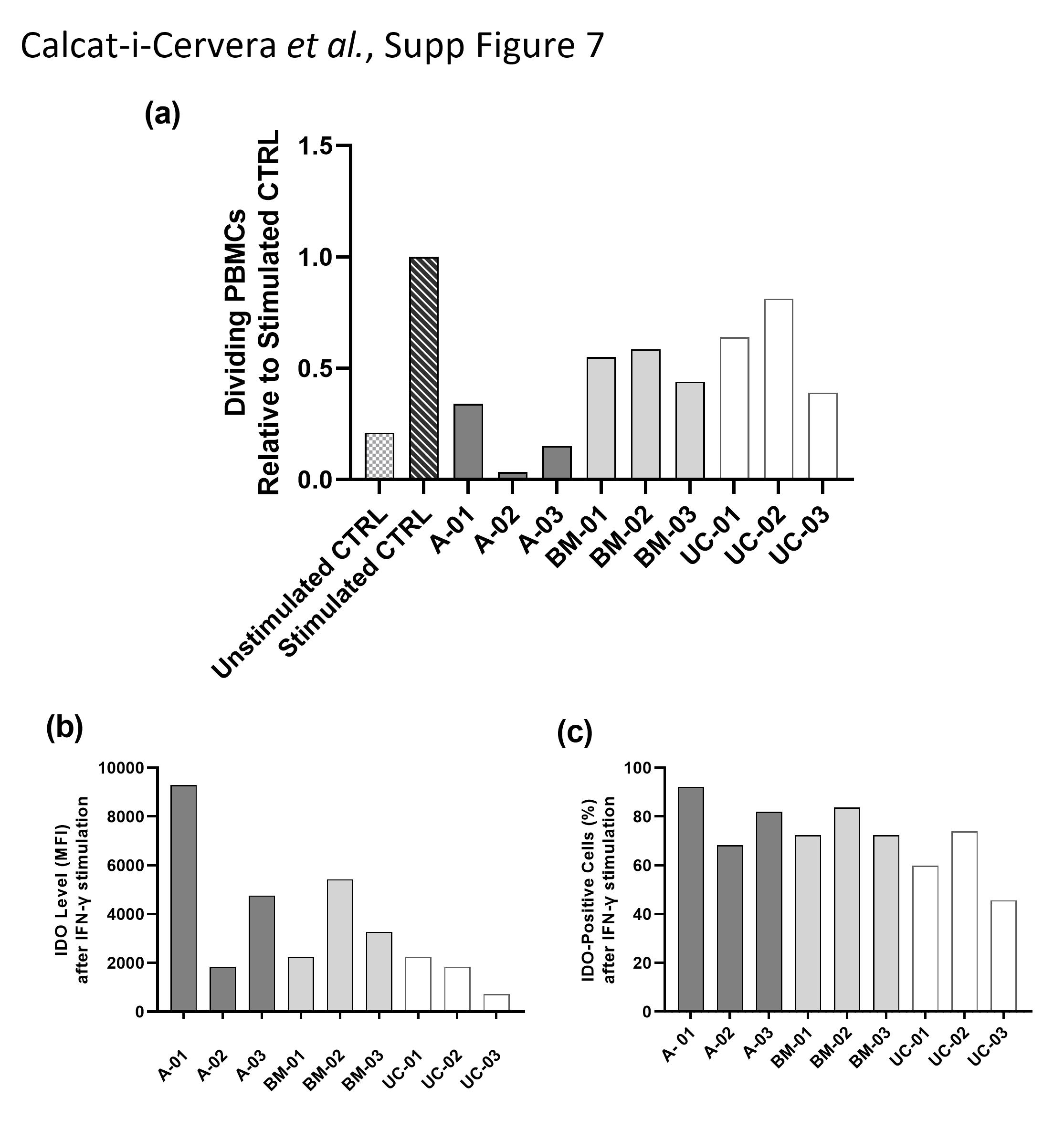

Supplement: Supplementary file 7 — Additional file 7: Supplementary Fig. S7. Donor-by-donor breakdown of MSC immunomodulatory capacity.Individual values of PBMC proliferation co-cultured with MSCs in the presence of PHA, where each bar represents the relative value in relation to PHA-stimulated PBMCs cultured alone.MFI of IDO intracellular staining andpercentage of IDO-positive cells after 24h of IFN-γ stimulation,listed per donor. [file 13287_2023_3352_MOESM7_ESM.jpg]

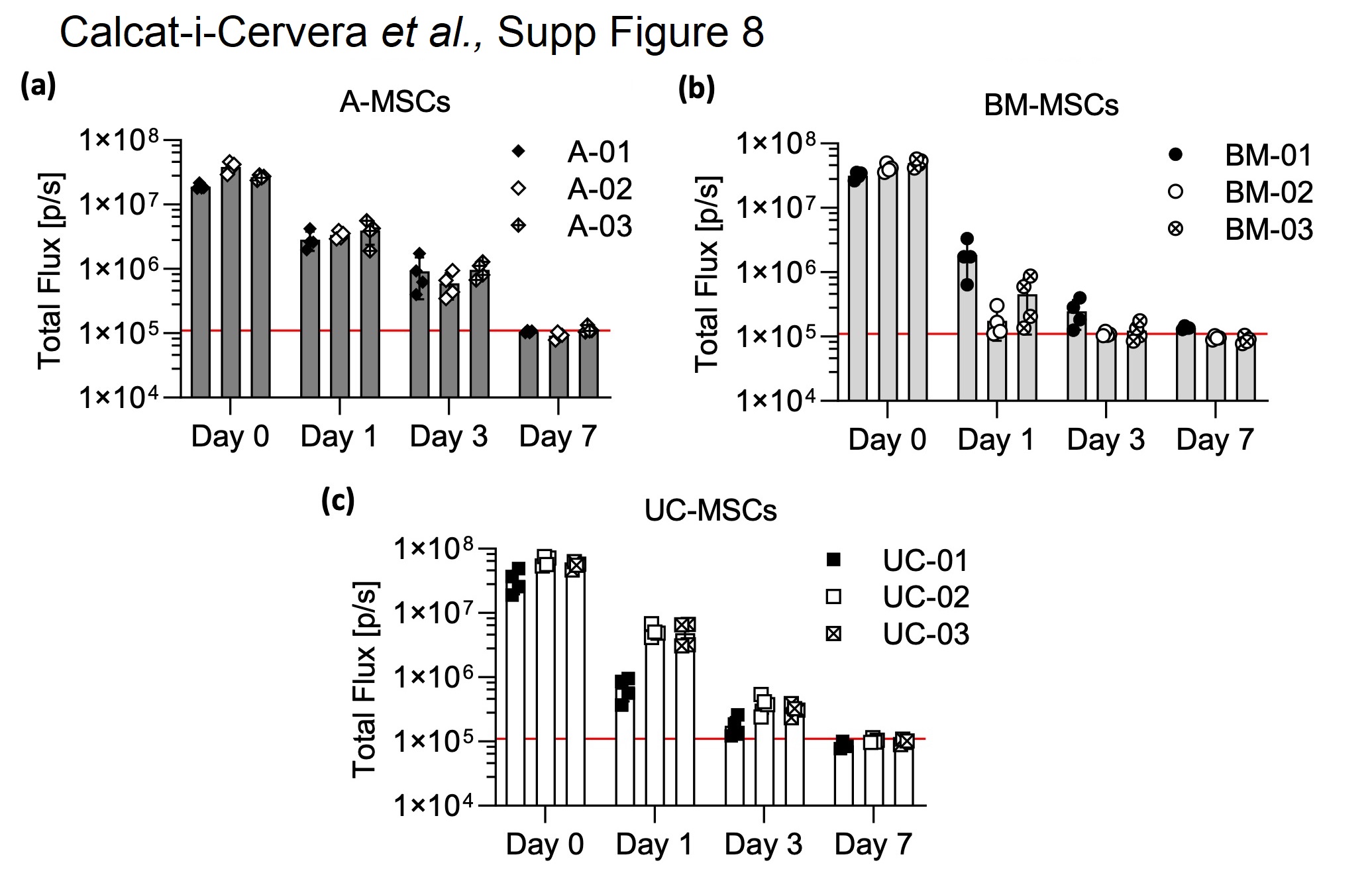

Supplement: Supplementary file 8 — Additional file 8: Supplementary Fig. S8. Donor-by-donor breakdown of the signal obtained from the in vivo imaging of MSCs in healthy C57BL/6 albino mice.Light outputas a function of timecoming from A-, BM-, and UC-MSCs. Data displayed as mean ± SD from N = 4 for each donor. The red lineis the background BLI signal emitted by naïve animalsthat did not receive any cells. [file 13287_2023_3352_MOESM8_ESM.jpg]
