# Supplementary material for: Harmonised culture procedures minimise but do not eliminate mesenchymal stromal cell donor and tissue variability in a decentralised multicentre manufacturing approach
Source: Stem Cell Res Ther. 2023 May 4;14:120. doi: 10.1186/s13287-023-03352-1 (PMC10161493; doi:10.1186/s13287-023-03352-1)
Supplement: Supplementary file 9 — Additional file 9: Supplementary Table S1. Detailed methodology used in each centre to characterise the immunophenotype of MSCs. [file 13287_2023_3352_MOESM9_ESM.docx]

**Additional file 1: Figures**

**Additional file 1: Figure 1**: **Cell Culture Harmonisation: Serum Screen**

(a-c) Population doubling times and (d) phase contrast images of three BM-MSCs donors showed that exclusively FBS A supported cell growth and fibroblast-like morphology. Therefore, further experiments were carried out using serum A.

(e) Flow cytometry confirmed the expression of positive surface antigens (CD90, CD73, CD105) and lack of negative markers (CD45, CD34, CD11b, CD19, HLA-DR) in two out of three populations grown with FBS A.

(f-i) BM-MSC cultures were induced to differentiate into adipocytes (+) while undifferentiated cultures served as control (-). Images of Oil Red O are shown in panel (i) and quantification of Oil Red O stain retention in panel (f). Both show an increase in lipid content in the majority of adipogenic differentiated cultures. (i) Osteogenic differentiated cultures showed presence of calcium in the extracellular matrix with Alizarin Red staining. (g) Quantification of extracted calcium from osteogenically differentiated BM-MSC showed more than 1 µg of calcium per well in all differentiated cultures. (h) Quantification of sulphated glycosaminoglycans (s-GAG) showed significantly increased levels in differentiated cultures (+), confirming their mesodermal differentiation abilities. Data displayed as mean ± SD, N=3. Two-Way ANOVA with Bonferroni’s multiple comparison corrections, * = p < 0.05, ** = p < 0.001, ** = p < 0.0001, **** = p < 0.00001. Pictures taken at 40X; scale bar 500 µm.

**Additional file 1: Figure 2. Cell Culture Harmonisation: Seeding Density**

Comparison between seeding density confirmed differences in cell source. Cumulative population doublings were calculated by culturing MSCs at 300 (empty symbols) and 3,000 cells/cm^2^ (filled symbols) in all three different sites. (a) A-MSC and (c) BM-MSC showed a rapid increase in cumulative doublings when seeded at a lower density versus at high density after the same period in culture. (e) UC-MSC conversely had increased cumulative doublings when seeded at higher density. (g) When comparing population doubling times, A-MSC and BM-MSC had prolonged kinetics when grown at 3,000 cells/cm^2^ whereas UC-MSC divided faster at 3,000 cells/cm^2^. (b,d,f) Representative phase contrast images of MSCs. Data displayed as mean ± SD, N=3. Pictures taken at 40X.

**Additional file 1: Figure 3.** Representative phase contrast images of MSCs in all sites at early (3-4 days) and late (5-10 days) stages of culture. Pictures taken at 100X; scale bar 200 µm.

**Additional file 1: Figure 4.** Biological comparison**:** donor-by-donor breakdown of doubling times, immunophenotype, differentiation results and phase contrast images of the differentiation.

Figures (a-c) show the individual doubling times per each donor in all sites. The three dots within a single donor represent the doubling times from three consecutive passages. Across laboratories, A- and UC- showed stable proliferation rates when looking at individual donors. Greater differences were seen in BM- in terms of donor-to-donor variability, although each donor behaved similarly regardless of manufacturing site. In terms of committing to mesodermal lineages, high variability of induction was seen across laboratories. Broadly, A- and BM- donors were able to undergo adipogenesis in two sites, apart from one particular donor that showed induction in all laboratories (d-f). Negligible levels of adipogenic differentiation were seen in UC-MSC cultures. Similarly, A- and BM-MSCs were able to undergo osteogenic differentiation in two out of three sites (g-i), albeit not all donors and at remarkable different rates; exclusively one UC-MSCs in one site showed moderate levels of osteogenesis. Assessment of surface antigen expression confirmed >95% levels of CD73, CD90 and CD105 in all donors across sites (j-l). However, two preparations of A-MSC showed higher than 2% levels of CD34 in two and CD45 in one site. Importantly, these were the same donors.

Data displayed as mean ± SD, N=3, n=3. One-Way ANOVA with Tukey’s multiple comparison corrections, * = p < 0.05, ** = p < 0.001, ** = p < 0.0001, **** = p < 0.00001.

**Additional file 1: Figure 5**. Representative phase contrast images of MSC at the end of the adipogenic (adipo) and osteogenic (osteo) differentiation procedure in comparison with undifferentiated cultures (ctr) in each site. Pictures taken at 100X; scale bar 200 µm.

**Additional file 1: Figure 6**. Angiogenic and wound healing properties of MSCs listed by donor. (a-c) Number of tubules (a), junctions (b) and closed loops (c) generated by each donor. Data expressed as a fold-change of the positive control; mean ± SD, n = 3. (d-f) Differential angiogenic proteomic profile detected for A- (d), BM- (e) and UC- (f) MSCs. Data expressed as fold change of the internal reference spots. (g) Wound closure induced by each donor per cell source at 8 and 24 hours. Data displayed as mean ± SD, n = 3. Two-Way ANOVA with Tukey’s multiple comparison corrections, * = p < 0.05, ** = p < 0.001, ** = p < 0.0001, **** = p < 0.00001. # Significance relative to negative control.

**Additional file 1: Figure 7.** Donor-by-donor breakdown of MSC immunomodulatory capacity

(a) Individual values of PBMC proliferation co-cultured with MSCs in the presence of PHA, where each bar represents the relative value in relation to PHA-stimulated PBMCs cultured alone. (b) MFI of IDO intracellular staining and (c) percentage of IDO-positive cells after 24h of IFN-γ stimulation,listed per donor.

**Additional file 1: Figure 8**. Donor-by-donor breakdown of the signal obtained from the in vivo imaging of MSCs in healthy C57BL/6 albino mice. (a-c) Light output (flux) as a function of time (days) coming from A- (a), BM- (b), and UC- (c) MSCs. Data displayed as mean ± SD from N = 4 for each donor. The red line (1.1 x 10^5^ p/s) is the background BLI signal emitted by naïve animals (n = 4) that did not receive any cells.

**Additional file 1: Tables**

Additional file 1: Table 1. Detailed methodology used in each centre to characterise the immunophenotype of MSCs

|  | **GALWAY** | **HEIDELBERG** | **LIVERPOOL** |
| --- | --- | --- | --- |
| **Instrument** | FACS Canto II (BD Biosciences) | FACS Canto (BD Biosciences) | FACScalibur (BD Biosciences) |
| **FACS Buffer** | 2% FBS in 1X DPBS, sterile filtered | 0.4 % bovine serum albumin BSA, 0.02 % sodium azide NaN3 in PBS, pH 7.4 |  |
| **Blocking** |  | 4°C for 5 minutes with FcR blocking reagent (Miltenyi Biotec, 130-059-901) |  |
| **Antibodies** | BD Biosciences StemFlow Human MSC Analysis Kit (BD Biosciences, 562245) | anti-CD44 (APC Cy7, Biolegend, 103028), anti-CD73 (PE, Biolegend 344004), anti-CD90 (APC, BD Biosciences, 559869, ), anti-CD-105 (PE-Cy7, Biolegend, 304016, ), anti-CD34 (APC, BD Biosciences 555824), anti-CD45 (PE-Cy7, Biolenged 304016), anti-HLA-DR (APC Cy7, Biolegend, 307618). | anti-CD11b (APC, Miltenyi Biotec, 130 113-793), anti-CD19 (APC, Miltenyi Biotec, 130-113-727), anti-CD34 (APC, Miltenyi Biotec, 130-113-738), anti CD44 (APC, Miltenyi Biotec, 130-113-893), anti CD45 (APC, Miltenyi Biotec, 130-113-676), anti CD73 (APC, Miltenyi Biotec, 130-097-945), anti-CD90 (APC, Miltenyi Biotec 130-117-534), anti CD105 (APC, Miltenyi Biotec, 130-099-125), anti-HLA-DR (APC, Miltenyi Biotec, 130-113-960), IgG1 mouse isotype (APC, Miltenyi Biotec, 130-113-758), or IgG2 mouse isotype (APC, Miltenyi, 130-113-831) |
| **Viability dye and concentration** | Draq7^TM^ (1:750 in FACS buffer) (BioStatus, DR70250) | Sytox Blue viability dye (1:2000 in FACS Buffer) (Invitrogen Life Technologies, S34859) |  |
| **Events recorded** | > 10^4^ | > 10^4^ | > 10^4^ |

**Additional file 1: Information**

**Materials and Methods**

1. **Serum Screen**

Three batches of FBS from different suppliers were tested to source a serum that supported the growth of MSCs from each tissue source in adequate amounts to service the complete project. Three already isolated BM-MSC donors were used, and we measured their proliferation rates, immunophenotype, and trilineage differentiation potential. Proliferation and immunophenotype were performed as described previously in the Materials and Methods section.

For adipogenic differentiation, confluent cultures were treated with adipogenic induction media, which consisted of high glucose Dulbecco’s modified eagle medium (HG-DMEM, SigmaAldrich, D5796) supplemented with 10% of each FBS respectively and 1% penicillin-streptomycin (PS, Gibco, 15140-122), 1 µM dexamethasone (Merck, D4902), 10 µg/mL insulin (Sigma, 11376497001), 200 µM indomethacin (Merck, I7378) and 500 µM 3-Isobutyl-1-Methyl-Xanthine (MIX, Merck, I7018)) during 3 days and subsequently with adipogenic maintenance media (HG-DMEM, 10% of each FBS, and 1% PS) for 1 day in three repeating cycles. At the completion of the last cycle, cells were incubated in maintenance media for 7 days. Control cells were maintained in regular culture media. Detection of intracellular lipid accumulation was achieved by staining the cultures with 3% Oil Red O (Sigma, O0625) in de-ionised water (6:4) after fixation with 10% neutral buffered formalin (Sigma-Aldrich, HT501128). Harris Modified Haematoxylin (Sigma-Aldrich, HHS-16) was used to counterstain before brightfield imaging at 4X on an Olympus BX43 microscope fitted with an HD Chrome camera (1/.8”) and a 0.5x C-mount adapter. For quantitative analysis, 99% isopropanol (Sigma-Aldrich, I9516) was used to extract the Oil Red O-stained lipids that were then quantified in a multimode plate reader via absorbance at 490 nm (Victor X3, Perkin Elmer).

For chondrogenic differentiation, harvested BM-MSCs were transferred to screw capped microcentrifuge tubes at a concentration of 2 x 10^5^ cells and centrifuged at 100 g for 5 minutes in a swing out rotor to generate cell pellets. Negative differentiated pellets were cultured with incomplete chondrogenic media (ICM: HG-DMEM supplemented with 100 nM dexamethasone, 50 µg/mL ascorbic acid 2-phosphate (Sigma, A8960) 40 µg/mL L-proline (Sigma, P0380), 1X ITS+ media supplement (insulin, transferrin, selenous acid, linoleic acid, bovine serum albumin), 1 mM sodium pyruvate,1% PS) while positive differentiated pellets were cultured with complete chondrogenic media (CCM: ICM supplemented with 10 ng/ml transforming growth factor β3 (TGF-β3, R&D Systems, UK). Media changes were performed every other day for 21 days. The level of chondrogenesis was assessed by measuring the sulphated glycosaminoglycans (s-GAG) present in each pellet using the DMMB assay and normalising between cultures by DNA content measured using PicoGreen^TM^ Quant-iT Kit (ThermoFisher Scientific, P11496) as per the manufacturer’s instructions.

For osteogenic differentiation, 80% confluent cultures were treated with osteogenic induction media, which consisted oflow glucose Dulbecco’s modified eagle medium (LG-DMEM, SigmaAldrich, D6046) supplemented with 10% of each FBS, 1% PS, 100 nM dexamethasone, 100 µM ascorbic acid 2-phosphate (Sigma, A8960), and 10 mM ß-glycerophosphate (Sigma, G9422). Control cells were maintained in regular culture media. Media changes were performed twice per week for 17 days. At the end of this period, cultures were washed with PBS and treated with 0.5 M HCl (Sigma, 1090581000) to collect the cell layer. The cell suspension was then incubated overnight at 4 °C under agitation, centrifuged to discard cell debris and the calcium present in the supernatant quantified using the Stanbio Calcium CPC liquicolour kit (Stanbio via ThermoFisher, 0150250) as per the manufacturer’s instructions. Representative brightfield images of the cultures were taken after fixation in 95% ice cold methanol and staining for calcium deposits with 2% Alizarin Red S (Merck, A5533). Images were taken as described for adipogenic cultures.
